# Multi-environment evaluation and stability analysis of early-maturing drought-tolerant tropical maize (*Zea mays* L.) hybrids using AMMI and GGE models

**DOI:** 10.3389/fpls.2026.1847825

**Published:** 2026-09-10

**Authors:** Chelang’at Sitonik, MacDonald Bright Jumbo, Dan Makumbi, Patrick Okori, James Mwololo, Hapson Mushoriwa, Mateete Bekunda, Irmgard Zeledon, Boddupalli M. Prasanna

**Affiliations:** 1 Department of Horticulture, College of Agricultural and Life Sciences, University of Wisconsin, Madison, WI, United States; 2 International Crops Research Institute for the Semi-Arid Tropics (ICRISAT), Matopos Research Station, Bulawayo, Zimbabwe; 3 Maize Program, International Maize and Wheat Improvement Center (CIMMYT), Nairobi, Kenya; 4 International Institute of Tropical Agriculture (IITA), c/o AVRDC- The World Vegetable Centre, Arusha, Tanzania; 5 Sustainable Intensification Program, International Institute of Tropical Agriculture (IITA), Ibadan, Oyo State, Nigeria

**Keywords:** drought tolerance, genotype stability, grain yield, multi-environment trials, tropical maize, yield adaptability

## Abstract

**Introduction:**

Adaptability and stability analysis play a key role in genotype selection under Genotype x Environment Interaction (GEI) for identification of suitable genotypes adapted to target environments.

**Methods:**

The current study analyzed GEI for stability and adaptability of drought-tolerant three-way hybrids using the additive main effect, multiplicative interaction (AMMI) and Genotype main effect, and genotype x environment interaction (GGE) models. Grain yield data used for the analysis was recorded from the trial conducted during 2018 and 2019 for two (wet) seasons, at six test environments; with three environments in Iringa district, two environments in Kongwa district (Dodoma region), and one environment in Kiteto district (Manyara region) using alpha lattice design. The field and agronomic management of the trial was standard at all test environments; data collected from environments that were not affected by drought were analyzed as optimal while that from environments which were slightly affected by drought were analyzed as moderate stress and the rest from severely drought affected sites were analyzed under severe stress.

**Results:**

Differences for grain yield were not significant (P<0.05) between maize hybrids analyzed under optimal conditions but significantly different when analyzed under moderate and severe stress conditions. Grain yield heritability estimates of 0.7 and 0.6 under optimal and moderate drought conditions, respectively, indicate strong genetic control of grain yield. Several hybrids outperformed commercial checks by up to 20% under drought stress. Grain yield for the 10 top performing hybrids ranged from 8 to 7.4; 6.7 to 4.6 and 3.1 to 0.9 t ha-1 under optimal, moderate and severe stress, respectively, which was better than SAWA, the drought tolerant check. The genotype main effect (G) and environment main effect (E) were highly significant (p<0.001) for grain yield; however, G × E effects were not significant. Ranking sites based on power of discriminating genotypes and representativeness, NGUMBI was the highly discriminating site, while IGULA was the most representative. MLALI and KIHOROGOTA were identified as the core test sites within the second quadrant.

**Discussion:**

Based on AMMI and GGE models, we identified stable and widely adapted hybrids and those with specific adaptation for deployment in semi-arid regions of Tanzania.

## 1 Introduction

Maize (*Zea mays* L.) is the third most important crop globally after wheat and rice (Erenstein et al., 2022; Mtyobile, 2021). Maize sustains the economic stability and food security of several households in Sub-Saharan Africa (SSA) due to its high grain yield potential and wide adaptability (Awata et al., 2019). Maize productivity and production in SSA need to increase to feed the rapidly growing population projected to be approximately 2.1 billion by 2050 (United Nations, 2017; Rezende et al., 2019). However, maize production in SSA has failed to keep pace with demand owing to biotic and abiotic factors (Cairns et al., 2021; Njeri et al., 2017). Biotic factors (which include maize lethal necrosis, gray leaf spot, turcicum leaf blight, maize streak virus, and fall armyworm) and abiotic stresses (such as drought and poor soil fertility) are major constraints that significantly reduce maize yield (Beyene et al., 2016). Furthermore, limited availability or access to quality seed of improved varieties in SAA contributes to low yields. These factors affect maize production at the farm level, resulting in low average yield (1.1 t ha^-1^), and this can be worse, mainly in drier regions such as the Manyara and Dodoma regions of Tanzania. These areas, which are classified as semi-arid, receive approximately 600 mm average annual rainfall; consequently, seed producers and sellers do not have the incentives to invest there due to the high probability of crop failure.

Therefore, the development, identification, and deployment of improved maize hybrids or varieties with drought tolerance (Rezende et al., 2019; Gebre et al., 2023) and good agronomic traits are the prime objectives for promoting food security and livelihoods of communities living in these marginal areas of Tanzania. Early-maturing drought-tolerant maize hybrids provide a potential solution to mitigate climate challenges, especially in areas where rain season is short and inadequate.

Crop researchers usually assess genotypes across different agro-climatic environments to determine their performance and adaptability prior to the commercial release. Genotype × environment interaction (GEI) and stability analysis in multi-environment trials (METs) are critical components for consideration by maize researchers when evaluating, selecting, and recommending crop varieties. GEI is a function of the interaction effect of the genotypes and test environments. Evaluating, selecting, and recommending genotypes for production in a particular environment are often complex, considering the challenge that arises from confounding effects of GEI, especially under conditions of biotic and abiotic stresses. When environmental conditions are divergent, crossover GEI or rank changes occur, further complicating genotype evaluation (Balestre et al., 2009). Therefore, evaluation of newly developed varieties across diverse environments (sites and seasons) is fundamental to estimate the magnitude of genotype × environment (G × E) and for cultivar recommendation with broader or narrow adaptation, and yield stability.

The major widely used models for stability analysis in previous studies include the following: the joint regression (Eberhart and Russell, 1966), which estimates genotype characteristics in a linear response to environmental fluctuations (Memon et al., 2023) but is not able to delineate the environments into few mega-environments; and the additive main effects and multiplicative interaction (AMMI) model (Zobel et al., 1988; Gauch and Zobel, 1996; Gauch and Zobel, 1997), which is a hybrid model incorporating analysis of variance (ANOVA; for additive component) and principal component analysis (PCA; for multiplicative component) to achieve a greater understanding of GEI and its causes and consequences. However, AMMI is incapable of identifying the close relationship between high mean performance and stability. To resolve this, genotype × environment interaction (GGE) biplot analysis is another commonly used multivariate model for describing data structure in crop improvement programs, which considers genotypic main effects plus GEI effects (Yan and Kang, 2003; Gauch et al., 2008; Neisse et al., 2018). It is also useful for determining the which-won-where pattern of the multi-environment trial data, thereby identifying high-yielding and stable genotypes. This model ranks environments based on the power to discriminate genotypes and identify representative test environments (Yan and Kang, 2003). It is a routine practice by researchers to use these models to explain GEI and analyze the performance of genotypes and test environments. Test sites that discriminate genotypes and are representative of the mega-environments provide crucial information about the genotypes and environments useful during assignment of genotypes to suitable production environments. Although drought-tolerant maize hybrids have been previously evaluated in METs and analyzed for their adaptation and stability using AMMI and GGE models (Rezende et al., 2019); these hybrids were medium-maturing hybrids, so they were not suitable for areas with annual rainfall of 600 mm or less. In the light of the above background, the objectives of this study were i) to identify the best-performing, drought-tolerant, three-way, early-maturing, hybrid maize evaluated in METs under optimal and stressed moisture conditions in Tanzania, ii) identify the best-adapted and stable maize hybrids suitable for specific environments and a wide range of adaptation using AMMI and GGE models, and iii) identify the best environments for evaluating experimental maize hybrids in Tanzania.

## 2 Materials and methods

### 2.1 Experimental sites (environments), design, and field management

**Figure 1:**
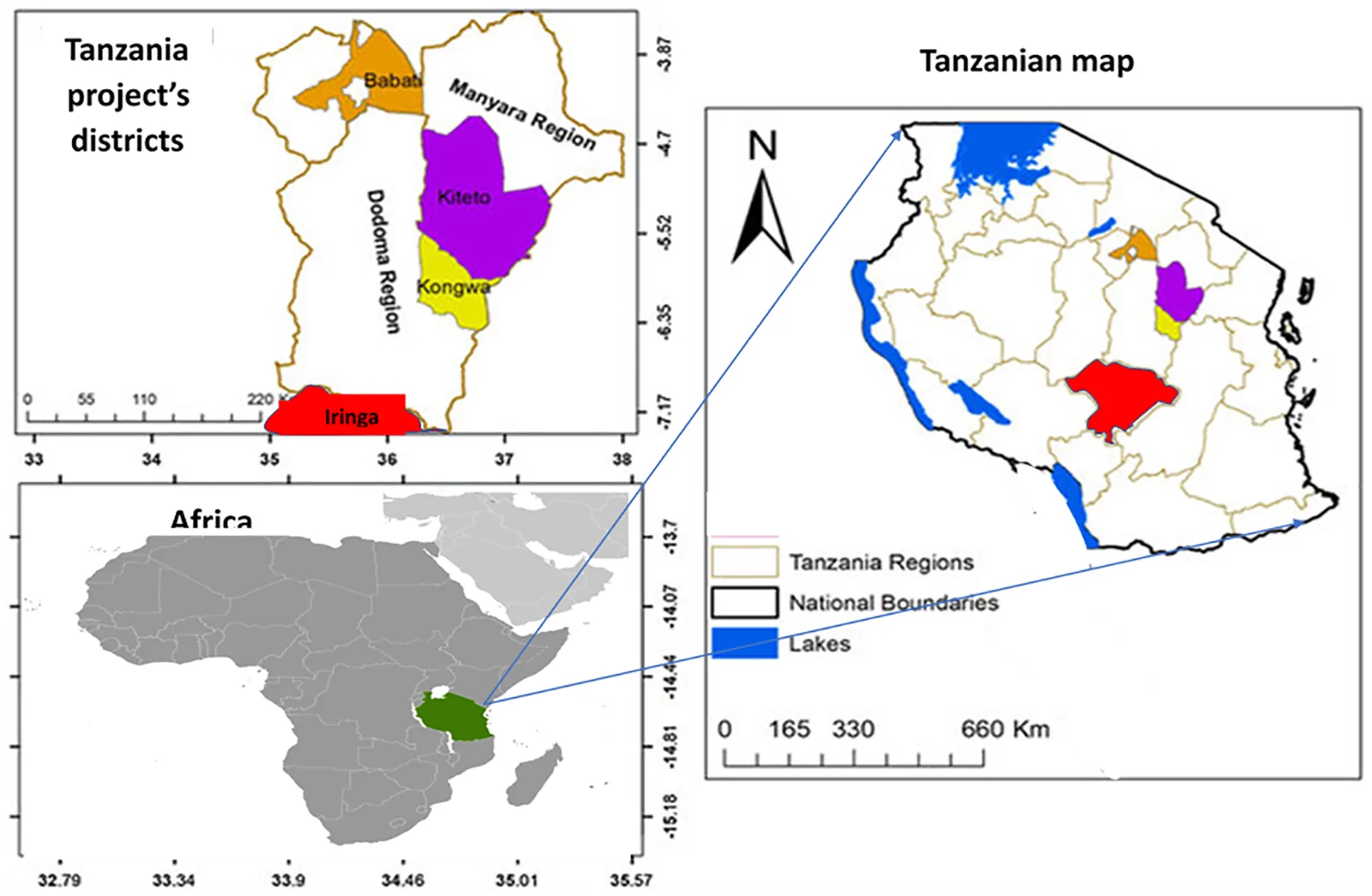
Map of Tanzania showing study area covering Kongwa, Kiteto, and Iringa districts.

This study was conducted during the wet season from December to April in the years 2018 and 2019 at six different test environments as per **Table 1**, **Supplementary Table 2A**, and **Figure 1**.

**Table 1:** Descriptions of the seven test environments used for evaluation of the 115 maize hybrids in the present study.

District	Location name	Annual rainfall (mm)	Latitude	Longitude	Elevation (masl^†^)	Soil type	Temperature (°C)	
							Max	Min
Iringa	Ismani	~700	−7.459	35.803	1,255	Luvisols	24.5	14.1
Iringa	Igula	<500	−5.24	36.279	1,203	Arenosols	22.8	13.6
Iringa	Kihorogota	~600	−7.449	35.81	1,134	Luvisols	27	15
Iringa	Ndolela	~500	−7.498	35.789	1,303	Interflavus	23	19
Kongwa	Mlali	~500	−6.269	36.765	1,230	Vertisol	29	17
Kongwa	Ng’humbi	~500	−6.317	36.826	1,363	Nitisols	23	18

† Meters above sea level.

A trial consisting of 107 three-way experimental maize hybrids, which were selected from a set of hybrids previously evaluated in MET (**Supplementary Table 2A**) and eight commercial checks, was formed with two replications using a 5 × 23 alpha lattice design (Patterson and Williams, 1976). Three-way hybrids were preferred for a broader genetic base to provide general adaptation compared to single-cross hybrids, considering the prevailing heterogeneous environmental conditions under smallholder farming. Each entry was planted on a 5-m-long, two-row plot, and two seeds were planted at each hill, with 0.25-cm spacing between hills within each row and 0.75-cm spacing between rows. The plants at each hill were thinned to one plant, 2 weeks after establishment (Njeri et al., 2017). Among the checks, DUMA43 was used as an early-maturing check, DSLH103 (SAWA) was used as a drought-tolerant and internal genetic gain check, WH507 was used as an internal genetic gain check, DK8031, PAN 4M-19, DHO4, and P2849W were used as commercial checks, and 3041 was used as a local check.

The trial was managed in all environments by applying good agronomic practices that included the application of basal fertilizers and top-dressing fertilizers according to recommended rates. The fields were kept free of weeds to avoid competition between plants and weeds for soil nutrients, moisture, and sunlight. The sites that received adequate rainfall without drought stress are referred to as optimal in this study. Although all the sites received similar good agricultural practices, some sites were affected by a dry spell during the plant growing period, and plants were exposed to moisture stress. Data from these sites were analyzed as random stress, and because it was moderate, the data from these sites were analyzed separately as moderate stress.

Some of the sites were severely affected by prolonged drought during the flowering time of the crop, resulting in significantly reduced yields. Data from these sites were analyzed as severe stress.

### 2.2 Trait phenotyping

Data were recorded on the following major agronomic traits during flowering and at harvest using defined protocols, as follows: anthesis date (AD), days to 50% tasseling and pollen shed within each plot; silking date (SD), days to 50% silking within each plot; anthesis-silking interval (ASI), determined as the difference between SD and AD; ear height (EH), measured in centimeters and determined as the distance from the base of the plant to the point of attachment of the top ear on a plant; plant height (PH), measured in centimeters and determined as the distance from the base of the plant to the height of the first tassel branch; plant aspect (PA), rated on a scale of 1 to 5, where 1 = short plant with uniform and short ear placement and 5 = tall plants with high ear placement or height (ear position); and ear aspect (EA), rated on 1–5 scale, where 1 = nice uniform ears with the preferred texture and 5=cobs with the undesirable texture. Field ear weight for each entry was recorded on a plot basis with cobs harvested from each plot 7.5 m^2^ to estimate grain yield adjusted to 80% shelling percentage and 12.5% grain moisture content, where representative samples of ears were shelled to determine percentage moisture using a Dickey John moisture meter at all locations and later expressed in t ha^-1^. The final traits considered for this study included grain yield (t ha^-1^), grain moisture (%), anthesis date, ASI, and plant stand, which are critical components for selection under semi-arid stress conditions.

### 2.3 Statistical analysis of phenotypic data

Collected phenotypic data were subjected to ANOVA for each site; after this, all data were merged; a pooled ANOVA was performed using META-R (v.5.0) (Alvarado et al., 2015), and Best Linear Unbiased Predictions (BLUPs) were generated by applying mixed model Y = Xβ + *Z*u + ϵ (Piepho et al., 2007), where y is the vector of observations; β and U are vectors of fixed and random effects, respectively; X and *Z* are the associated design matrices; and ϵ is a random residual vector. The random effects are assumed to be distributed as u ~ MVN (0; G) and ϵ ~MVN (0, R), where MVN(µ, V), and V denotes the multivariate normal distribution with mean vector µ and variance–covariance matrix V. The fixed effects can be estimated by Best Linear Unbiased Estimation (BLUE), while the random effects can be estimated by BLUP. Data from moderately drought-stressed environments and from severely drought-affected sites were analyzed separately but with all the genotypes represented at all sites.

Broad-sense heritability was computed from variance components (Hallauer et al., 2010) using META-R (v.6.03) (Alvarado et al., 2015), as follows:


$$H^{2}=\frac{\sigma_{G}^{2}}{\sigma_{G}^{2}+\frac{\sigma_{GL}^{2}}{e}+\frac{\sigma_{E}^{2}}{er}}$$

where $\sigma_{G}^{2},\;\sigma_{GL}^{2},\;\sigma_{E}^{2}$, *e*, and *r* are the genotype, genotype by location, residual components, number of environments, and number of replications, respectively.

### 2.4 Genotype × environment interaction and stability analysis

GEI effects detected in ANOVA were explored using AMMI (Zobel et al., 1988; Gauch and Zobel, 1997) and GGE biplot models to analyze GEI. AMMI and GGE biplot models were used to determine the effects of genotypes, environments, and their interaction. The genotypes were represented by entry numbers for easy visualization of output.

#### 2.4.1 Additive main effects and multiplicative interaction model

AMMI combines ANOVA and PCA for additive and multiplicative components using the following AMMI statistical model:


$$Y_{ijk}=µ+G_{i}+E_{j}+\sum_{k=1}^{m}\lambda_{k}\alpha_{ik}\delta_{jk}+R_{ij}+\epsilon ,$$

where *Y_ij_* is the value of *i*th genotype in *j*th environment; *μ* is the grand mean; *G_i_* is the mean of the *i*th genotype minus the grand mean; *E_j_* is the deviation of the *j*th environment from the grand mean; *λ_k_* is the singular value for PC axis *k*; *α_ik_* and *δ_jk_* are the PC scores for axis *k* of the *i*th genotype and *j*th environment, respectively; *R_ij_* is the residual; and *ϵ* is the error term.

AMMI stability value (ASV) was calculated for each hybrid to quantify stability and rank all genotypes as per Purchase et al. (2000) using the following formula:


$$ASV=\;\sqrt{{\left[\left(\frac{\text{SSIPCA}1}{\text{SSIPCA}2}\right)\left(\text{IPCA}1\right)\right]}^{2}+{\left(\text{IPCA}2\right)}^{2},}$$

where $\frac{\text{SSIPCA}1}{\text{SSIPCA}2}$ is the weight given to the first interaction principal component score (IPCA1) value due to its high contribution in the GE model, obtained by dividing the sum of squares of IPCA1 by the sum of squares of IPCA2; the IPCA1 and IPCA2 scores are the genotypic scores in the AMMI model. The larger the ASV, the more specifically adapted the genotype is to a certain environment, and the lower the ASV value (closer to zero), the lower the genotype’s interaction with the environment; consequently, the genotype is said to be more stable across environments.

Using the Metan R package (Olivoto and Lúcio, 2020), additive main effects and multiplicative interaction biplots were constructed across environments from the mean values of the hybrids and commercial checks. The relationships among genotypes and environments were illustrated based on biplots generated from the first PCA and the mean grain yields of the genotypes and environments.

#### 2.4.2 Genotype main effect and genotype × environment interaction biplot

GGE biplots were generated based on singular-value decomposition (SVD) of the first two principal components in R using the following model:


$$Y_{ij}-{µ}_{i}-\beta_{j}=\sum_{k=1}^{m}\lambda_{k}\alpha_{ik}{ϒ}_{jk}+\epsilon_{ij},$$

where *Y_ij_* is the performance of genotype *i* in environment *j*; *μ* is the grand mean; *β_j_* is the main effect of environment *j*; *k* is the number of principal components (PCs); *λ_k_* is the singular value of the *k*th PC; *α_ik_* and ϒ*_jk_* are the scores of the *i*th genotype and the *j*th environment, respectively, for PC*_k_*; and *ϵij* is the residual associated with genotype *i* in environment *j*.

The BLUPs for each hybrid across environments for the hybrids and the commercial checks were used for GGE biplot analysis. The relationship of GEI was represented by the “which‐won‐where” pattern. An environment-centered GGE biplot was generated to visualize associations among test environments and hybrids. An average environment coordination (AEC) was drawn to observe the mean performance and stability of the hybrids and to identify ideal test environments and hybrids.

The most stable and adapted genotypes were identified using stability parameters, coefficient of regressions (*b_i_*) = 1.0, and the mean of squared deviations from regression $\left(S_{di}^{2}\right)=0$ (Eberhart and Russell, 1966).

## 3 Results

### 3.1 Hybrid performance and variability

Results showed that differences between hybrids for grain yield were not significant (*p* < 0.05) when evaluated under optimal conditions but were significant under moderate and severe stresses (**Table 2**). The difference between the mean grain yield of 8.0 t ha^−1^ for the best-performing maize hybrid (CKH160197) compared to the mean grain yield of 6.7 t ha^−1^ for the best check DK8031 and the grand mean of 6.4 t ha^−1^, under optimal conditions, was significant. The mean grain yield of the best-performing hybrid (CKDHH170346) under moderate drought was 6.7 t ha^−1^, higher than the mean of the best commercial and genetic gain check DSLH103 (SAWA), which was 5.4 t ha^−1^, and was also above the grand mean of 5.1 t ha^−1^. The mean grain yield for the top-performing hybrid (CKH160236) under severe stress was 3.3 t ha^−1^, three times higher than the mean of the best commercial and genetic gain check, DSLH103 (SAWA), which had a mean of 1.1 t ha^−1^. Higher heritability estimates of 0.7 and 0.6 for grain yield were observed when hybrids were evaluated under optimal conditions and moderate stress conditions, respectively (**Table 2**).

**Table 2:** Mean performance for grain yield and agronomic traits of the top 10 hybrids from the 115 maize hybrids evaluated under optimal and drought conditions.

Name	₸Optimal	†MS stress env.	††SS env.	⸆AD (days)	⸆⸆ASI (cm)	±PH (cm)	±±EH (cm)	GM₸₸ (%)	PS††† (#)
	Grain yield (t ha^-1^)								
CKH160197	8	5.5	1.2	60	3	192	84	11.8	29
CKH160236	7.8	5.3	3.1	60	3.1	202	96	11.8	29
CKH160207	7.7	4.8	1.3	60	3.1	199	90	11.8	29
CKH160213	7.7	5.5	1.7	60	3.1	210	99	11.7	28
CKH160275	7.7	4.6	1.9	63	3.4	207	95	12	28
CKDHH170346	7.6	6.7	0.9	65	3.1	209	102	11.8	28
CKDHH170282	7.6	5.8	2.9	67	3.2	207	104	11.9	29
CKH160266	7.5	5.4	1.9	63	2.9	206	93	11.8	29
CKH160269	7.5	5.2	2.1	63	3.5	203	95	11.9	29
CKDHH170276	7.4	6.4	1.3	64	3.2	222	112	12	29
DK8031	6.7	4.5	1.3	63	3.1	217	106	11.6	28
PAN 4M-19	6.7	4.7	0.8	61	3.2	202	96	11.8	27
Local check: 3041	6.3	3.7	0.9	61	3.1	209	102	11.7	29
DUMA43	6.3	4.5	1.5	61	3.3	221	97	11.9	28
DSLH103 (SAWA)	6.2	5.4	2.4	63	3	195	88	11.7	27
DHO4	4.9	2.2	1.8	65	3.1	211	96	11.9	26
WH507	4.6	4	1.9	71	2.9	213	103	12.2	28
P2849W	3	1.6	1	68	3.1	182	79	11.8	19
Grand mean	6.4	5.1	3.4	62.2	3.1	204	94	11.8	28.4
LSD (0.05)	2.6	1.1	0.01	2.8	1	11	9	0.6	2.1
CV	20.4	19	38.5	3.2	69.3	6	8	10.9	8
# Replicates	2	2	2	2	2	2	2	2	2
SE (±)	0.99	0.75	0.81	1.96	2.17	11.37	7.68	1,28	2.28
Heritability	0.7	0.6	0.5	0.8	0.1	0.9	0.9	0.2	0.5

Data highlighted in gray color show hybrid selections under each key trait based on best checks and Least Significant Difference (LSD). *Significant at *p* ≤ 0.05; ^₸^Normal rain season without drought stress; ^†^Moderate drought stress during flowering; ^††^Severe drought stress environments during flowering; ^†††^Plant stand per plot; ^₸₸^Grain moisture; ⸆Anthesis date; ^⸆^Anthesis-silking interval; ^±^Plant height; ^±±^ Ear height.

Another critical agronomic trait associated with drought tolerance is the ASI. Results from this study showed that moderate ASI was observed among the 10 top-performing hybrids, ranging from 2.9 to 3.4, and differences between these top hybrids with top-performing check for this trait were not significant (*p* < 0.05) (**Table 2**). Furthermore, the flowering time is another critical trait when developing maize hybrids targeting areas with a short rainfall season. Results for flowering time for these hybrids showed that the 10 top-performing hybrids had flowering time (anthesis) ranging from 60 to 67 days with an average of 62.5 days, and significant differences between hybrids and checks were observed (*p* < 0.05) (**Table 2**). This is within the expected range and comparable or better than that of most of the checks. Heritability for flowering time was higher at 0.8, implying that this trait is highly heritable and strongly genetically controlled.

### 3.2 Stability and adaptability analysis using AMMI model

The combined ANOVA for the hybrids evaluated across environments based on the AMMI model is presented in **Table 3**. The results showed that genotype main effect (G) and environment main effect (E) were highly significant (*p* < 0.001) for grain yield; however, G × E effects were not significant. The test environments contributed 70.14% of the total variation in the sum of squares for grain yield. G × E contributed 20.33% of the total variation, where 9.52% was due to the genotypic main effect and 10.8% to the interaction effect.

**Table 3:** AMMI analysis of variance of grain yield of drought-tolerant hybrids across six environments.

Source of variation	df	Sum of squares	Mean square	% explained	Total variation explained (%)
Environments (E)	5	2,854.2	570.83688***	70.141	70.141
Genotypes (G)	114	387.48	3.39891***	9.522	79.663
Interaction (G × E)	570	827.58	1.45189 ns	20.337	100
PC1	118	267.1	2.26359***	33.271	33.271
PC2	116	217.49	1.87494***	27.092	60.363
PC3	114	173.43	1.52129***	21.603	81.966
PC4	112	141.39	1.26244*	17.612	99.578
PC5	110	3.389	0.03081*	0.422	100
PC6	108	0	0	0	100
Residuals	689	1,005.2	1.459	0	0

*Significant (p < 0.05); ***Highly Significant (p < 0.001).

The first two PCs explained 60.36% of the total variability (**Table 3**). The PCs are ordered according to decreasing contribution to variation. The first four PCs were significant and explained 99.59% of the G × E interaction (**Table 3**). Since the G × E effects were not significant, it was not necessary to perform further analysis to compute stability values and stability parameters.

**Figure 2:**
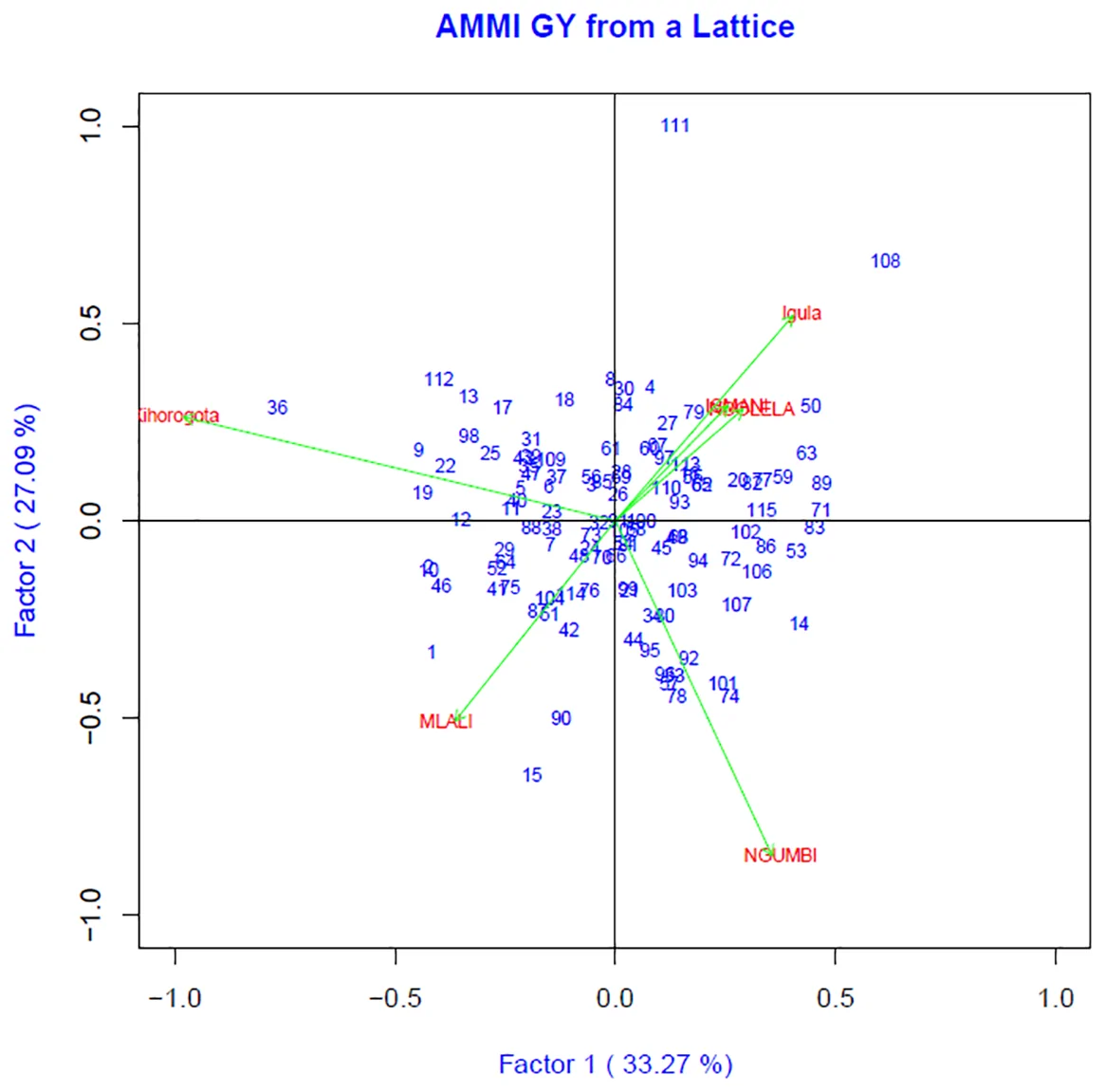
AMMI plot showing the performance and association of the maize hybrids at six environments. AMMI, additive main effects and multiplicative interaction.

The AMMI model biplot based on the first two PCs and the genotype means was generated to illustrate the performance and association of the genotypes (**Figure 2**). Several genotypes were clustering near the origin, implying that their yield across environments was stable and widely adaptable. Some genotypes, however, performed better in specific environments (**Figure 2**), indicative that these genotypes are adapted to specific environments. Commercial hybrids DH04 (Entry 108) and P2849W (Entry 111) are specifically adapted to environments Igula, Ndolela, and Ismani; test hybrids CKDHH170097 (Entry 1), CKH160177 (Entry 90), and CKDHH170112 (Entry 15) are specifically adapted to Mlali; and hybrid CKDH160038 (Entry 36) is specifically adapted to Kihorogota. Therefore, they will not be stable and cannot perform well if grown in environments other than where they are specifically adapted.

### 3.3 Stability and adaptability analysis using GGE biplot

**Figure 3:**
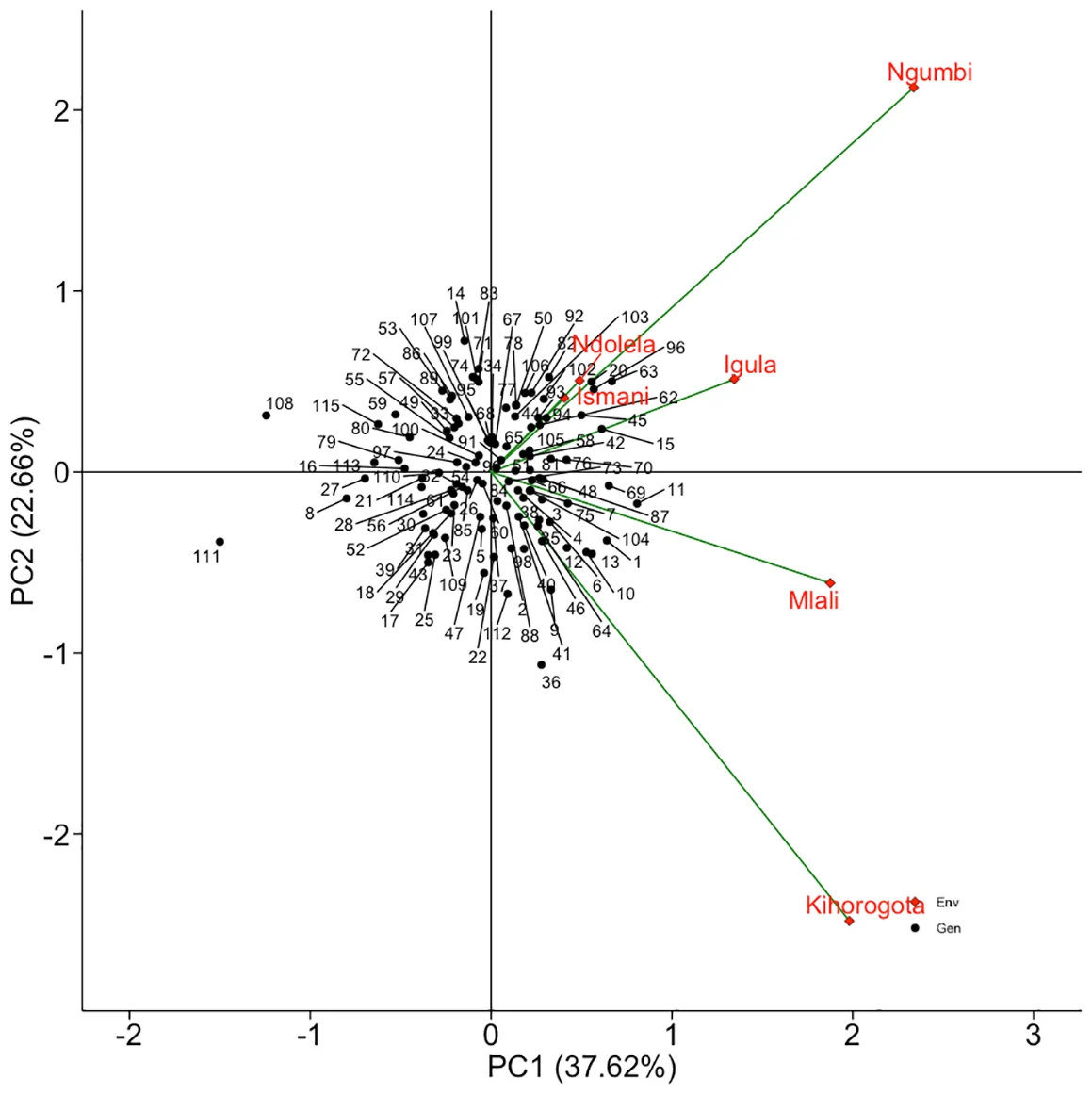
Interrelationships between the test environments indicating which test locations are better than others for evaluating maize hybrids.

**Figure 4:**
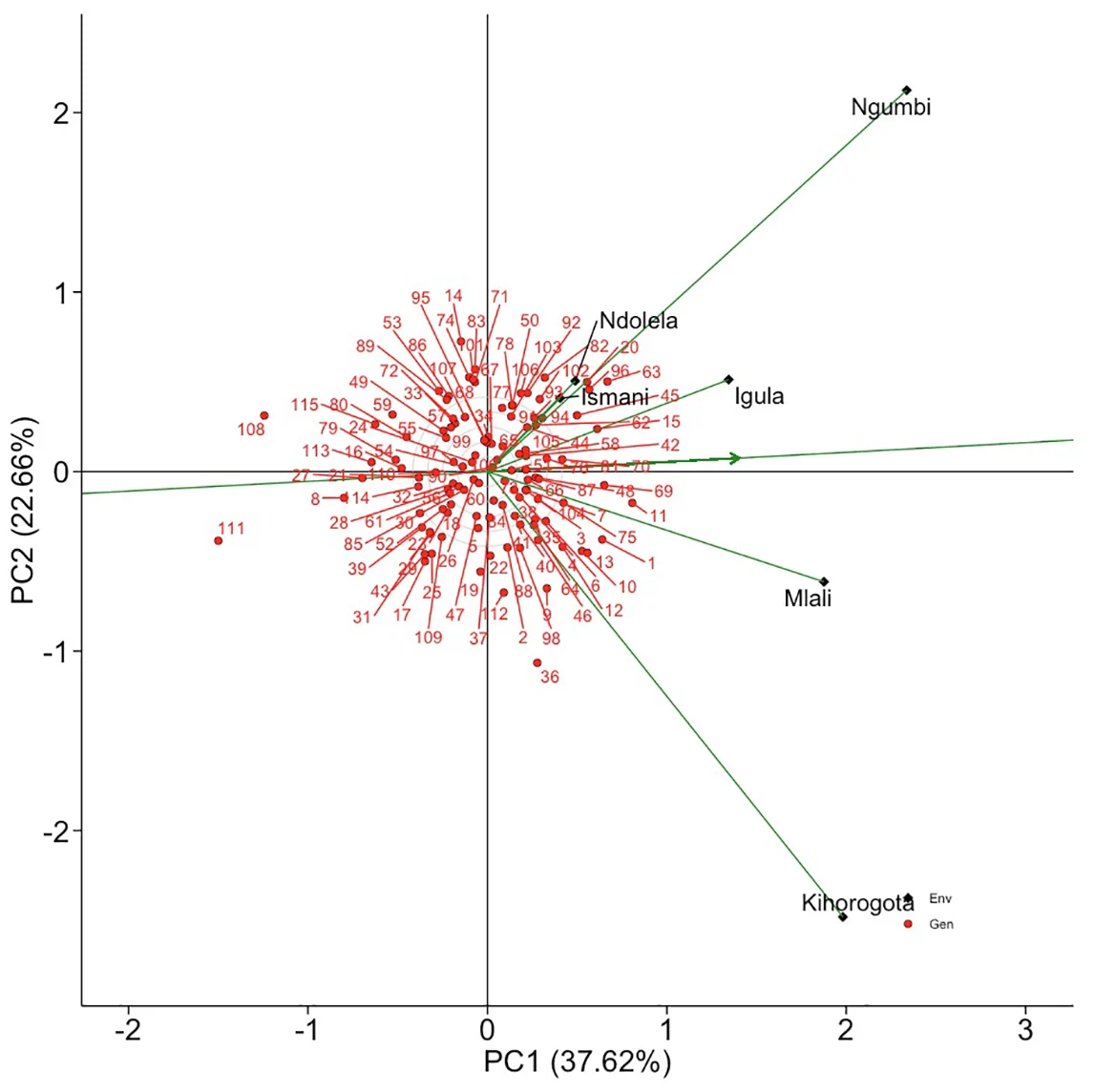
The vector view of the GGE biplot based on environment-focused singular-value partitioning showing the discriminating power and representativeness of the test environments. GGE, genotype × environment interaction.

**Figure 5:**
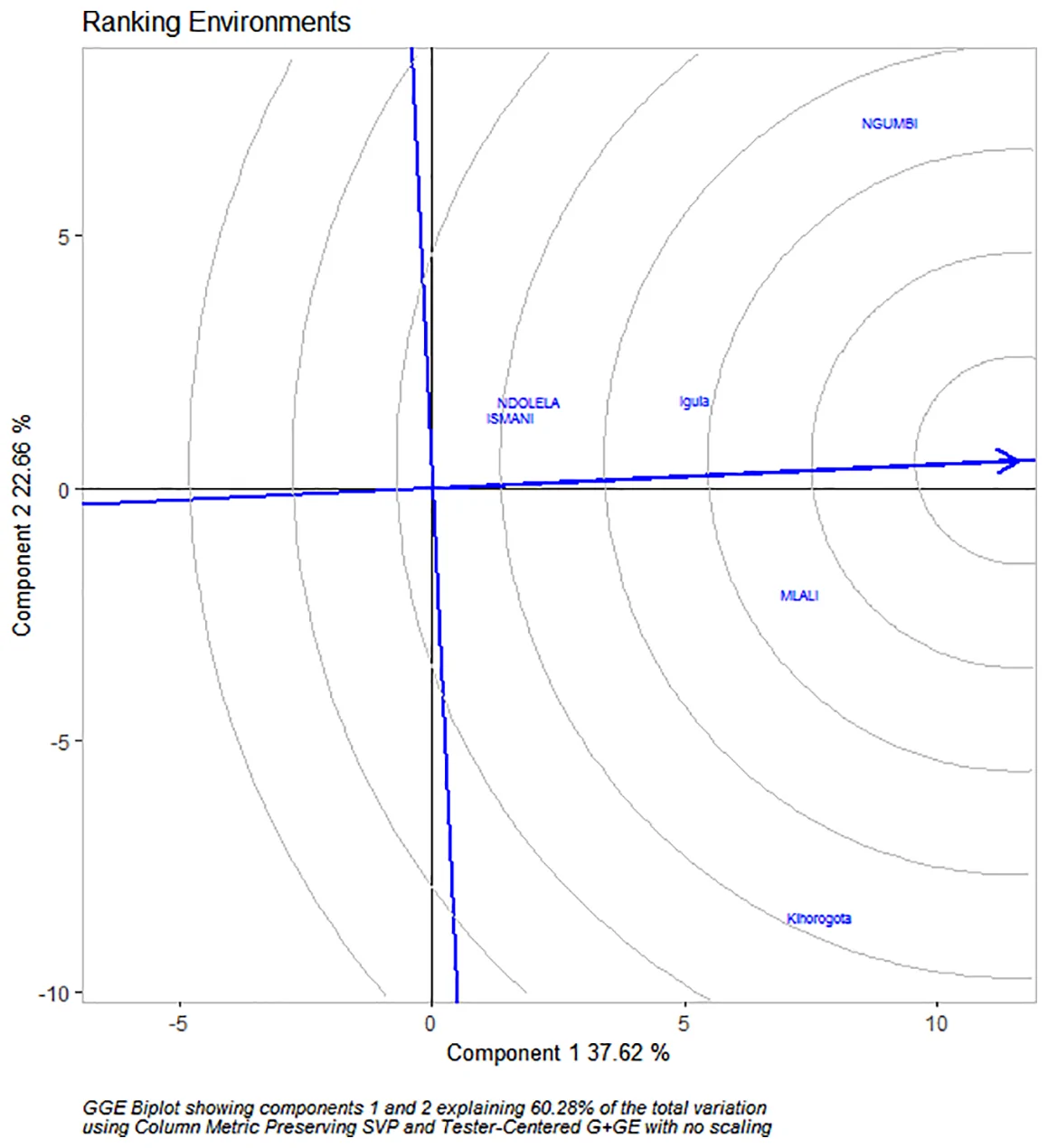
Biplot for comparison of all environments with the ideal environment, constructed based on environment-centered and environment-focused singular-value partitioning.

**Figure 6:**
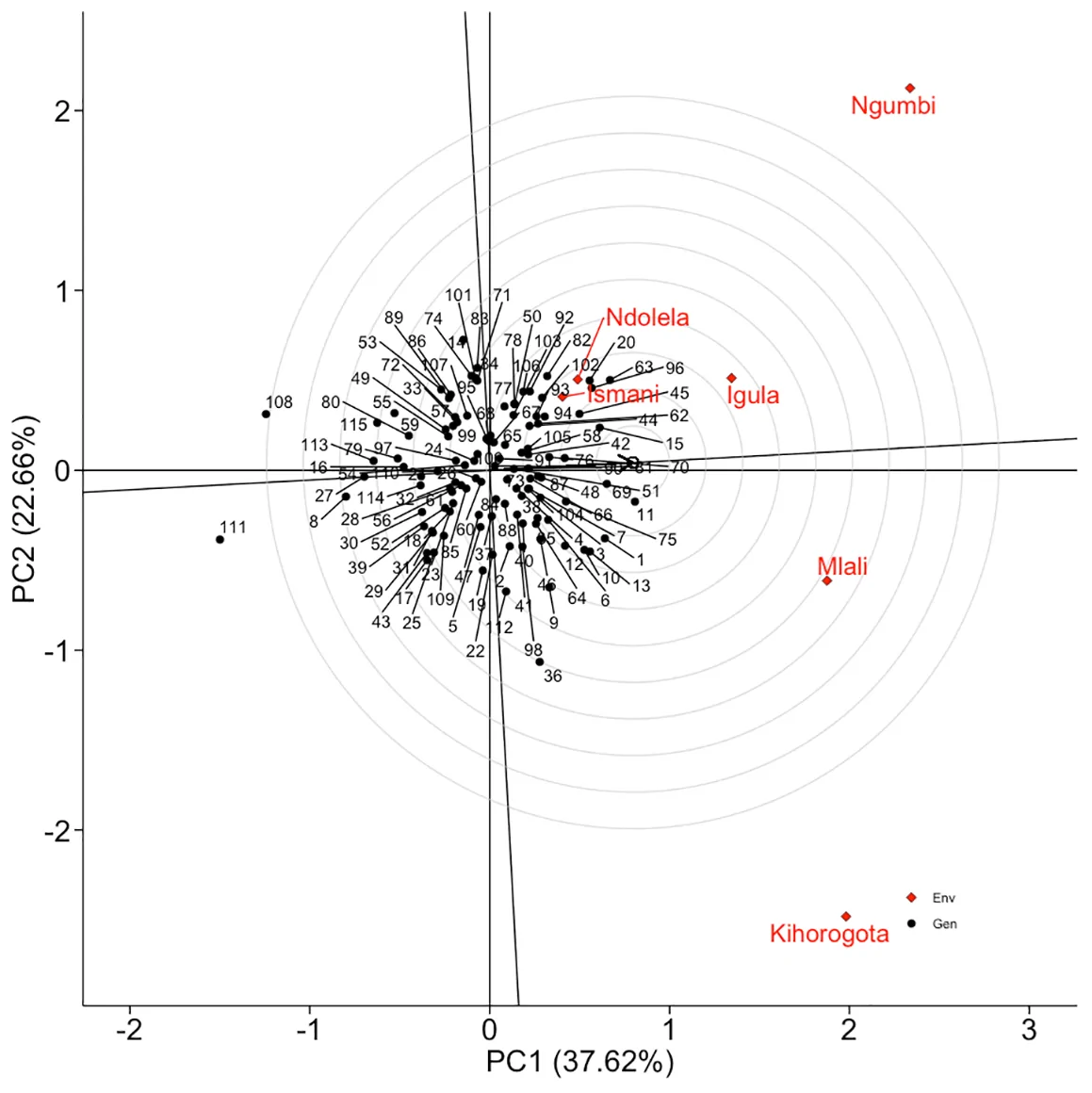
Biplot showing comparison of all genotypes with the ideal genotype, constructed based on environment-centered and genotype-focused singular-value partitioning.

**Figure 7:**
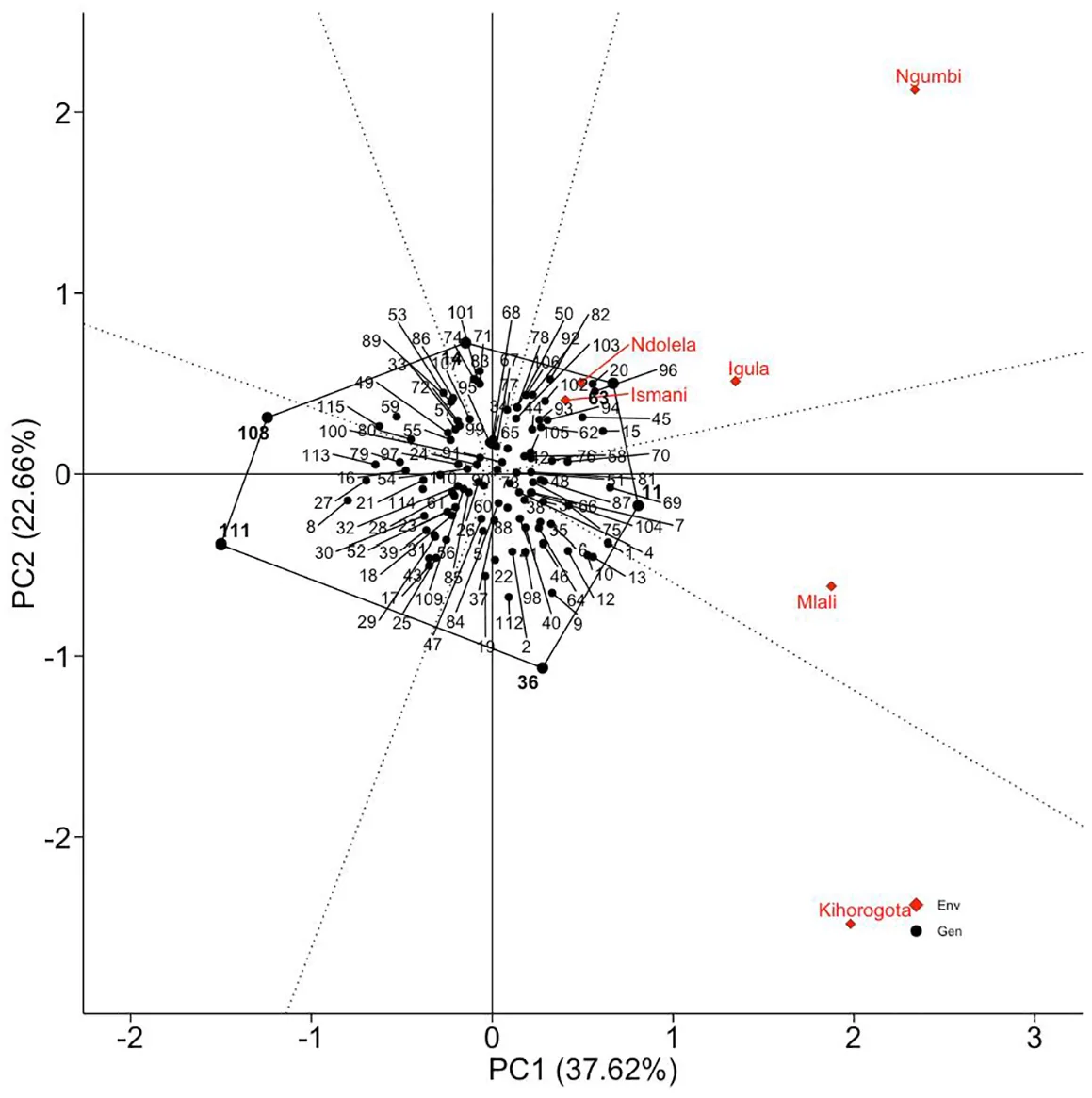
The “which–won–where” view of the GGE biplot under each mega-environment constructed based on environment-centered and symmetrical singular-value partitioning.

**Figure 8:**
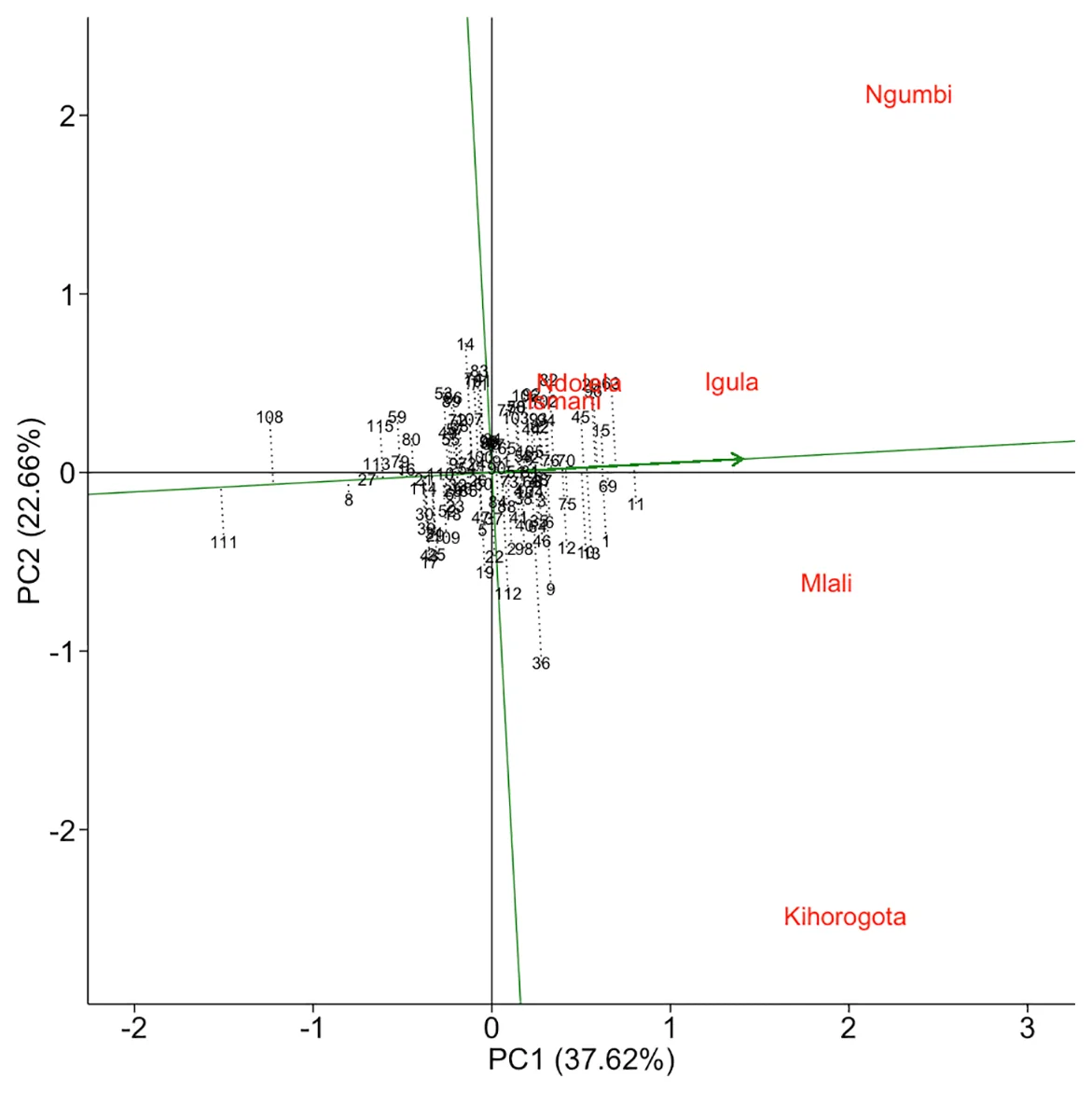
The “mean vs. stability” view of the genotypic main effects and genotype × environment interaction (GGE) biplot view of selected drought-tolerant maize hybrids evaluated at six environments.

**Figure 9:**
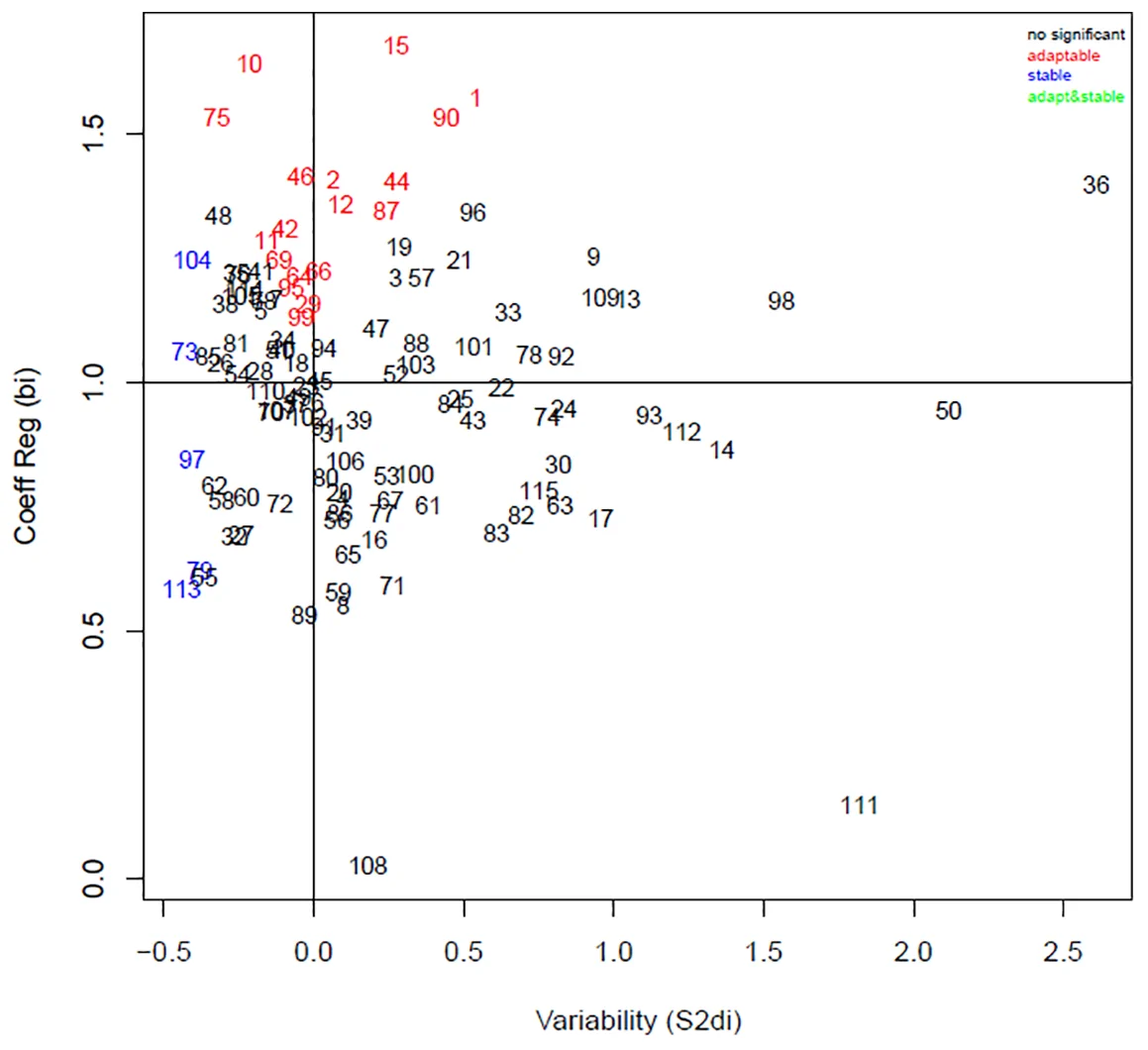
Mean performance and stability of hybrids, coefficient of variability (X-axis), and coefficient of regression (Y-axis). All numbers inside the biplot represent genotypes (maize hybrids).

The GGE biplot presented in **Figure 3** shows the interrelationships between the test locations. The environment vectors connect the biplot origin to the markers of the locations. The GGE biplots based on the first two principal components (PC1 and PC2) explained 60.28% of the total variation, with PC1 and PC2 explaining 37.62% and 22.66%, respectively (**Figures 3**–**9**). **Figure 4** provides a description of discrimination and representativeness. Results show that Ng’humbi has the longest vector, while Ismani has the shortest vector, implying that Ng’humbi discriminates genotypes much better than other sites, providing more informative results that would support good selection decisions for best-performing genotypes; Ismani is a poor site that would provide performance information that may not be very informative to make accurate selection decisions. However, an arrow close to the horizontal axis in the first quadrant indicates the position where the representative site should be; therefore, Igula, being closer to this arrow, can be considered the representative site. Results presented in **Figure 5** show the ranking of environments with reference to the ideal environment represented by the arrow vector. Igula, among the target environments, had a smaller angle with reference to the arrow vector, therefore considered an ideal environment for evaluating maize hybrids. Hybrids were ranked to find the ideal genotype, and results (**Figure 6**) show that CKH160156 (Entry 69), CKDHH170108 (Entry 11), and CKDHH170112 (Entry 15) were close in proximity to the small circle located on the AEC abscissa that represents the ideal genotype, so they are considered ideal hybrids.

#### 3.3.1 Which-won-where pattern

GGE biplot analysis results are presented in **Figure 7**, with a polygon view of the biplot displaying which hybrid performed well in which environment based on the which-won-where pattern. A polygon was drawn by connecting hybrids that were furthest from the biplot origin such that all hybrids were enclosed within the polygon. Based on the test locations used in this study, the results revealed six environment points in a polygon with different best-performing hybrids (**Figure 7**). Hybrids CKDHH170108 (Entry 11), DH04 (Entry 108), P2849W (Entry 111), CKDH160038 (Entry 36), CKDHH170111 (Entry 14), and CKH160150 (Entry 63) were on the vertices of the polygon; therefore, they performed well in those environments as assigned in the respective quadrants, implying that they have specific adaptation. Most of the hybrids were clustering around the center, implying that those hybrids may not perform well in the specific environments in the different quadrants, but on average, they can still perform in many environments, indicating that they have general adaptation.

#### 3.3.2 Mean vs. stability of the evaluated hybrids

The mean and the stability of the hybrids were visualized by drawing an AEC view of the GGE biplot using genotype-focused scaling (**Figure 8**).

The stability of the hybrid was measured by their projections onto the AEC coordinates (i.e., vertical line). The top-ranking hybrids according to their projections onto the AECs or axes were CKH160150 (Entry 63), P2849W (Entry 11), and CKDH160038 (Entry 36). Hybrids with greater absolute length of projection from the AEC coordinate have a greater contribution to GEI and are less stable across the test environments (Yan et al., 2007).

### 3.4 Summary of key hybrids based on stability and adaptability metrics

Several hybrids were stable because of their short projections onto the AEC coordinate. Further analysis was performed to classify hybrids based on the Eberhart and Russell coefficients (adaptable, stable, and no significant) using the AMMI model that utilizes variability and the coefficient of regression option (**Figure 9**). Five hybrids—CKH160191 (Entry 104), CKH160160 (Entry 73), CKH160184 (Entry 97), CKH160163 (Entry 79), and WH507 (Entry 113)—were stable across environments, while 18 hybrids were identified as adaptable. Interestingly, hybrids that are both adaptable and stable were not identified (**Figure 9**).

## 4 Discussion

### 4.1 Hybrid performance under contrasting environments

Maize production under smallholder farming in SSA is highly dependent on rainfall as one of the major contributing factors, which is subject to frequent fluctuations with recurring droughts in recent years, especially in the face of climate change. Development, identification, and deployment of early-maturing, climate-resilient, high-yielding, and stable maize hybrids are crucial for commercialization in environments prone to drought.

In this study, results showed that the mean of grain yield of the top 10 best-performing maize hybrids under optimal, moderate stress, and severe stress conditions was 7.7, 5.2, and 1.8 t ha^−1^, respectively, higher than the mean of the best commercial checks, which was 5.6, 3.8, and 1.8 t ha^−1^ under the same respective conditions, which was consistent with findings from previous studies (Rezende et al., 2019). Higher yields observed for several drought-tolerant experimental hybrids compared to the top best commercial checks and the drought-tolerant and internal genetic gain check, SAWA (**Table 2**), demonstrate that these hybrids have good potential for adaptation in these semi-arid test environments and are good candidates for release and commercialization. Some hybrids were adapted to specific environments and were less stable. In addition to the observed yields, anthesis was also observed in this study (**Table 2**), and results showed that most of the 10 top-performing hybrids had a lower number of days to reach 50% flowering, with an average of 62.5, which was close to the experimental mean, but the majority were between 60 and 61 days. Lower numbers of days of flowering are desirable for short-duration hybrids to fit in areas where the rain season is short, with the range being 60 to 69 days. In addition to flowering time, another critical trait associated with drought tolerance is the ASI. The ASI is the time gap between male flowering (tassel shedding pollen) and female flowering (silk emergence). In tropical maize, a short ASI (ideally 0 to 2 days) is a primary selection trait for drought tolerance because it ensures effective synchronization between pollen availability and silk receptivity (Bolaños and Edmeades, 1996). The ASI observed in the results for the 10 top-performing hybrids had an average of 3, statistically not significantly different from the desirable interval range.

### 4.2 Genotype × environment interaction, stability, and adaptability analyses

A wide variation was observed in the present study, especially the large sum of squares presented in **Table 3**, associated with the environment, indicating that the test environments were diverse. However, GEI was not significant, implying that it had little effect on the performance of hybrids across the six test environments. Environment accounted for 70.14% of the total variation, followed by GEI, accounting for 20.33% (**Table 3**). Similar results were reported by Badu-Apraku et al. (2011), where environment accounted for 83.4% of the total variation in grain yield, while GEI and G contributed 11% and 1.5%, respectively. Environmental effects have significant importance in contributing to the performance of genotypes that result in genotype performance variations within and between environments. This is consistent with results from previous studies (Badu-Apraku et al., 2011; Mafouasson et al., 2018) reporting that the test environments contribute more variation in genotype performance compared to other sources of variation. These studies further showed that the environment contributes a large proportion to yield stability.

Estimates of heritability present the proportion of trait variation attributable to differences between genotypes after accounting for variation explained by known environmental factors. This study showed high-to-moderate heritability when the maize hybrids were evaluated under optimal and drought stress (**Table 2**). The high heritability estimates observed indicated that much of the phenotypic variance was genetically controlled, and grain yield is a highly heritable trait and can be effectively improved through phenotypic selection in this set of hybrids. These estimates were within the range of those reported from previous studies (Cairns et al., 2013; Yuan et al., 2019). Furthermore, higher heritability is expected when genotypes are evaluated under optimal conditions compared to stress conditions. This is because optimal conditions allow the maximum expression of genetic potential, whereas drought or stress conditions create environmental “noise” (higher residual variance) that masks genetic differences (Nasser, 2020).

AMMI biplot showed the distribution of environments from low to high yields in different quadrants (**Figure 2**). In each quadrant, hybrids clustering around the center are stable, while those far from the center showed specific adaptability, implying that they perform better in those areas within each quadrant (Yan et al., 2007).

### 4.3 Evaluation of test environments: representativeness and discriminatory power

#### 4.3.1 AMMI model

The relationship among the environments based on the first two PCs (**Figure 2**) illustrates that Igula is similar to Ndolela and Ismani, indicating that they have a closer association or similarity of the environments for the evaluation of the genotypes. Therefore, we can discard any of the environments without losing the precision of the result since closely associated environments are expected to produce similar results. Therefore, these three environments can be delineated under a similar mega-environment (Yan and Kang, 2003). Igula had the longest distance from the origin of the biplot; therefore, it was the most discriminating environment for the hybrids, while Kihorogota, Mlali, and Ng’humbi are the three other environments. This information is useful for the development, deployment, and recommendation of similar hybrids to several locations under the same mega-environment.

#### 4.3.2 GGE biplot

The relationship among environments is approximated by the cosine of the angle between their vectors (Yan and Tinker, 2006). When the angle is less than 90°, the correlation coefficient between two environments is positive. An obtuse angle indicates a negative correlation, and a right angle shows that the test environments are not correlated. The angles among four environments (Igula, Ndolela, Ismani, and Ng’humbi) were<90° (**Figure 4**), indicating a high relationship among these test environments; therefore, any of these environments could be dropped to reduce the cost of field evaluation without losing the precision of the information. The smaller the angle within 90°, the more highly correlated the environments. Ndolela and Ismani are redundant sites because they had small angles showing a strong positive association of these environments. The test locations were clustered into two groups (Igula, Ndolela, Ismani, and Ng’humbi) in the first quadrant, and Mlali and Kihorogota in the second quadrant (**Figures 3**, **5**).

The length of the vector, which approximates the standard deviation (SD) within each environment, is a measure of the environment’s ability to discriminate the hybrids, i.e., the ability of an environment to discriminate among hybrids based on their performance (Yan and Kang, 2003). The longer the length of the vector, the higher the discriminating power of that environment. As shown in **Figure 4**, Ng’humbi had the longest vector; therefore, it was the most discriminating environment, while Ismani, with the shortest vector, was the least discriminating site. Shorter environmental vectors indicated that the specific environments were not strongly associated with the longer vector environments. The short-vector environments were less informative and provided little or no information on the performance of the tested hybrids compared to the longer-vector environments.

The cosine of the angle between the vector of a test environment and the average environment axis (AEA) estimates the genetic correlation between environments and illustrates the representativeness of the test environment (Yan and Tinker, 2006). The smaller the angle between the AEA and the environment, the greater the representativeness of the environment. As presented in **Figure 4**, Ng’humbi was found to be highly representative.

Furthermore, **Figure 5** shows the ideal environment, whereby the center of the concentric lines is used to measure the distance between each environment and the ideal environment. The small circle in the biplot represents the average environment, which is defined by the average coordinates of all the test environments (**Figure 5**). The nearer an environment is to the ideal environment, the more desirable that environment is for selecting superior genotypes. Therefore, Igula, Ng’humbi, and Mlali are ideal environments for the drought-tolerant hybrids.

### 4.4 Evaluation of test environments: representativeness and discriminatory power

#### 4.4.1 Which-won-where pattern

The “which‐won‐where” patterns divided the biplot into different sectors, indicating that different hybrids perform differently in these environments. Therefore, crossover GEI exists to suggest the possibility of dividing the test environments into mega‐environments (Gauch and Zobel, 1997; Yan and Kang, 2003). The genotypes at the vertex in each sector represent the highest-yielding hybrid in the environment that is within that particular sector (Yan and Tinker, 2006; Yan et al., 2007). **Figure 7** (polygon) shows six sectors with hybrids: CKH160150 (Entry 63), CKDHH170108 (Entry 11), CKDH160038 (Entry 36), P2849W (Entry 111), DH04 (Entry 108), and hybrid CKDHH170111 (Entry 14) as the vertex hybrids. Genotypes CKH160150 (Entry 63), CKDHH170108 (Entry 11), and CKDH160038 (Entry 36) located at the vertex had the longest vectors, so they are the top-yielding in their environments. These hybrids were, therefore, among the most responsive genotypes to environments in their respective directions compared to other hybrids.

None of the test environments were in the sector where genotypes P2849W (Entry 111), DH04 (Entry 108), and CKDHH170111 (Entry 14) are vertex hybrids, implying that these hybrids were the least performing in some or all of the test environments.

Hybrids located closer to the origin of the biplot were more stable than the vertex hybrids. Perpendicular lines were then drawn to each side of the polygon starting from the biplot origin, dividing the biplot into different sectors.

The environment group within each sector and the cultivar at the polygon’s boundary characterize the mega-environment (Yan et al., 2007; Yuan et al., 2019). The test environments in the present study were in three sectors, with Ndolela, Ismani, Igula, and Ng’humbi situated in the same sector, while Mlali and Kihorogota were in different sectors (**Figure 7**). This pattern suggests that the test environments may consist of three different mega-environments. Therefore, during the selection of test sites, one should strategically consider this pattern to avoid selecting sites that are in the same mega-environment, and some hybrids will be selected based on general performance across these mega-environments (stable hybrids), while others will be based on specific adaptation suitable in specific mega-environments.

To avoid random crossover GEI, mean performance and yield stability are useful when evaluating genotypes in diverse environments. Therefore, stability testing is crucial for the identification of an ideal genotype through METs. An ideal genotype is one that has the highest yield and is the most stable across environments. We identified the desirable hybrids that had closer proximity to the ideal genotype represented by the small circle located on the AEC abscissa (**Figure 6**). The axis of the AEC abscissa, or AEA, is the single-arrowed line that passes through the origin of the biplot and the average environment and points to the higher mean yield across environments. Accordingly, hybrids are ranked along the AEA based on mean performance. An arrow pointing to it indicates that it had the highest yield compared to all the hybrids evaluated in the tested environment and is considered stable across environments. The most desirable hybrids with proximity to the ideal genotype were CKH160156 (Entry 69), CKDHH170108 (Entry 11), and CKDHH170112 (Entry 15). Superior hybrids were separated from those that were below average grain yield based on the grand mean of the experiment by a vertical line. Therefore, entries on the left side had below-average mean yield, and the hybrids on the right side of the vertical line (AEC, double arrow) had above-average mean grain yield.

Alternatively, other approaches (Eberhart and Russell, 1966) can be used to assess the adaptability and stability of genotypes. According to Eberhart and Russell, a genotype is adaptable when the coefficient of regression (*b_i_*) is close to 1 (*b_i_* = 1). Genotypes are stable when the coefficient of variability (*S*^2^*^di^*) is near zero (*S*^2^*^di^* = 0). Most hybrids (18 hybrids) evaluated had higher *b_i_* values, indicating better adaptability of these hybrids to target environments.

#### 4.4.2 Stability and adaptability of hybrids

Most seed producers and suppliers are interested in commercializing hybrids that demonstrate stable adaptation, considering that the seed business involves a lot of investment costs, including promotion, which could incur significant cost. Therefore, maize hybrids that are stable if well adopted by farmers can be grown for a long time, which would provide a good opportunity for seed companies to continue marketing such hybrids in the target market segment. This study performed the Eberhart and Russell coefficients (adaptable, stable, and no significant) using the AMMI model and identified five hybrids (**Figure 9**)—CKH160191 (Entry 104), CKH160160 (Entry 73), CKH160184 (Entry 97), CKH160163 (Entry 79), and WH507 (Entry 113)—that were stable across environments, while 18 hybrids were identified as adaptable. This implies that these hybrids can attract the interest of the seed producers to market them in these target semi-arid environments, as the identified hybrids provide the genetic stability that ensures reliability, securing long-term revenues for investors and best performance for farmers.

### 4.5 Complementarity and integration of AMMI and GGE outcomes

ANOVA results generated from the AMMI model showed varying outcomes with significant G and E effects but non-significant G × E effects (*p* < 0.001) (**Table 3**), implying that even though the environmental variations were present, their influence had little importance on the varying performance of the genotypes at different test sites. This observation may need further investigation by increasing the test seasons to collect more information that would support the analysis to justify the switch in ranking and the change in the performance of genotypes observed at different sites, as observed in the analysis based on BLUPs (**Table 2**). Further analysis using the GGE model helps examine more closely the genotypes’ performance and the characterization of the test environments, ranking and identifying ideal environments and those that are best for discriminating performance of genotypes. Similarly, the GGE model (**Figure 8**) has further identified top-performing hybrids, ideal hybrids, hybrids that performed well in specific environments, and less stable hybrids according to projections onto the AEC or axis (Yan et al., 2007).

## 5 Conclusion

This study evaluated early-maturing maize drought-tolerant hybrids in METs under optimal and stress conditions in Tanzania to identify superior hybrids. Stability and GEI were analyzed using AMMI and GGE models to highlight which genotypes produce high, consistent yields across a wide range of conditions versus those suited for specific, localized environments. Furthermore, these analyses dissected complex interactions to separate the genetic effect (G), the environmental effect (E), and their interaction (GEI) to reveal whether a crop variety’s performance shifts dramatically depending on where it is grown.

The study identified over 10 experimental maize hybrids that showed superior yield and agronomic traits under optimal and stress conditions compared to the best commercial checks as well as internal genetic gain checks. These varieties are recommended for release for cultivation in these agro-ecologies, which usually receive limited annual rainfall.

GEI was not significant but explained 20.33% of the variation in the performance of the hybrids, and this was demonstrated by the switching of ranking by hybrids under optimal conditions, moderate stress, and severe stress. Further analysis using GGE biplot and AMMI models led to the identification of maize hybrids that performed well across environments (general adaptation) while others responded well to specific environments (specific adaptation). Based on results from both models, several hybrids demonstrated general adaptation, implying that they can be grown in wide agro-ecologies in Tanzania. Therefore, such hybrids are recommended for release and commercialization for wide adaptation. However, the models also identified other hybrids that responded better in specific environments; therefore, such hybrids have specific adaptation and should be recommended for release and commercialization targeting these specific agro-ecologies. Maize hybrids CKH160150 (Entry 63), CKDHH170108 (Entry 11), and CKDH160038 (Entry 36), identified in the analysis to be located at the vertex of the polygon, were the top-yielding hybrids in their specific environments assigned on the vertex, so they are recommended for release and cultivation targeting those specific environments.

The analysis further identified some hybrids as ideal genotypes, and these included CKH160156 (Entry 69), CKDHH170108 (Entry 11), and CKDHH170112 (Entry 15). Most of the hybrids evaluated showed good general adaptation, but the study did not find hybrids that were both adaptable and stable.

Ranking of environments for similarity and discriminating power showed that some environments were similar in terms of discriminating the genotypes; therefore, such information could help prioritize site selection by dropping some sites that would not provide additional value, thereby improving cost efficiency in conducting METs.

High-performing drought-tolerant hybrids identified from this study can offer a good solution for improving maize production in areas with similar agro-ecologies but facing recurrent drought challenges, therefore contributing significantly to food security in SSA.

Apart from generating information for variety release and commercialization, results from this study provide an opportunity for future follow-up studies to consolidate these findings by integrating more climate and environmental information through the application of geographic information system (GIS) models.

## Data Availability

The original contributions presented in the study are included in the article/**Supplementary Material**. Further inquiries can be directed to the corresponding author.
