# Supplementary material for: Multi-environment evaluation and stability analysis of early-maturing drought-tolerant tropical maize (Zea mays L.) hybrids using AMMI and GGE models
Source: Front Plant Sci. 2026 Sep 10;17:1847825. doi: 10.3389/fpls.2026.1847825 (PMC13601267; doi:10.3389/fpls.2026.1847825)
Supplement: Supplementary file 1 [file DataSheet1.pdf]

Supplementary table

Table 2A. Names of the maize hybrids and their pedigrees

Entry	Hybrid Code	Pedigree
1	CKDHH170113	(CML312/CML442)//CKDHL142086
2	CKDHH170114	(CML312/CML442)//CKDHL142979
3	CKDHH170115	(CML312/CML442)//CKDHL143014
4	CKDHH170274	(CKDHL0228/CML442)/CKDHL144004
5	CKDHH170277	(CKDHL0228/CML442)/CKDHL144005
6	CKDHH170282	(CKDHL0228/CML442)/CKDHL144010
7	CKDHH170329	(CKDHL0228/CML442)/CKDHL144084
8	CKDHH170367	(CKDHL0228/CML442)/CKDHL144122
9	CKDHH170273	(CML312/CML442)/CKDHL144004
10	CKDHH170276	(CML312/CML442)/CKDHL144005
11	CKDHH170284	(CML312/CML442)/CKDHL144011
12	CKDHH170313	(CML312/CML442)/CKDHL144072
13	CKDHH170316	(CML312/CML442)/CKDHL144075
14	CKDHH170328	(CML312/CML442)/CKDHL144084
15	CKDHH170346	(CML312/CML442)/CKDHL144108
16	CKDHH170366	(CML312/CML442)/CKDHL144122
17	CKH160195	(CML546/CZL0718)//(ZEWAc1F2-134-4-1-B-1-B*5/G16BNSeqC4-(F22x1)-2-3-1-B)-B-71-B-1-1-B-B-4

Table 2A. Names of the maize hybrids and their pedigrees

Entry	Hybrid Code	Pedigree
18	CKH160196	(CML540/CML539)//(((KU1403 x 1368)-7-2-1-1-B-B/CML444)-B-3-3-3-1-1/CL107252)DH10-B-2-B-1
19	CKH160197	(CML539/CML442)//(((KU1403 x 1368)-7-2-1-1-B-B/CML444)-B-3-3-3-1-1/CL107252)DH10-B-2-B-1
20	CKH160198	(CML545/CML548)//(((KU1403 x 1368)-7-2-1-1-B-B/CML444)-B-3-3-3-1-1/CL107252)DH10-B-2-B-1
21	CKH160199	(CZL0919/CZL0710)//(((KU1403 x 1368)-7-2-1-1-B-B/CML444)-B-3-3-3-1-1/CL107252)DH10-B-2-B-1
22	CKH160200	(CML541/CZL0718)//(((KU1403 x 1368)-7-2-1-1-B-B/CML444)-B-3-3-3-1-1/CL107252)DH10-B-2-B-1
23	CKH160201	(CML546/CZL0718)//(((KU1403 x 1368)-7-2-1-1-B-B/CML444)-B-3-3-3-1-1/CL107252)DH10-B-2-B-1
24	CKH160202	(CML540/CML539)//(((KU1403 x 1368)-7-2-1-1-B-B/CML444)-B-3-3-3-1-1/CL107252)DH10-B-2-B-2
25	CKH160203	(CML539/CML442)//(((KU1403 x 1368)-7-2-1-1-B-B/CML444)-B-3-3-3-1-1/CL107252)DH10-B-2-B-2
26	CKH160204	(CML545/CML548)//(((KU1403 x 1368)-7-2-1-1-B-B/CML444)-B-3-3-3-1-1/CL107252)DH10-B-2-B-2
27	CKH160205	(CZL0919/CZL0710)//(((KU1403 x 1368)-7-2-1-1-B-B/CML444)-B-3-3-3-1-1/CL107252)DH10-B-2-B-2
28	CKH160206	(CML541/CZL0718)//(((KU1403 x 1368)-7-2-1-1-B-B/CML444)-B-3-3-3-1-1/CL107252)DH10-B-2-B-2
29	CKH160207	(CML546/CZL0718)//(((KU1403 x 1368)-7-2-1-1-B-B/CML444)-B-3-3-3-1-1/CL107252)DH10-B-2-B-2
30	CKH160208	(CML540/CML539)//(((KU1403 x 1368)-7-2-1-1-B-B/CML444)-B-3-3-3-1-1/CL107252)DH10-B-2-B-3
31	CKH160209	(CML539/CML442)//(((KU1403 x 1368)-7-2-1-1-B-B/CML444)-B-3-3-3-1-1/CL107252)DH10-B-2-B-3
32	CKH160210	(CML545/CML548)//(((KU1403 x 1368)-7-2-1-1-B-B/CML444)-B-3-3-3-1-1/CL107252)DH10-B-2-B-3
33	CKH160211	(CZL0919/CZL0710)//(((KU1403 x 1368)-7-2-1-1-B-B/CML444)-B-3-3-3-1-1/CL107252)DH10-B-2-B-3
34	CKH160212	(CML541/CZL0718)//(((KU1403 x 1368)-7-2-1-1-B-B/CML444)-B-3-3-3-1-1/CL107252)DH10-B-2-B-3

Table 2A. Names of the maize hybrids and their pedigrees

Entry	Hybrid Code	Pedigree
35	CKH160213	(CML546/CZL0718)//(((KU1403 x 1368)-7-2-1-1-B-B/CML444)-B-3-3-3-1-1/CL107252)DH10-B-2-B-3
36	CKH160214	(CML540/CML539)//(((KU1403 x 1368)-7-2-1-1-B-B/CML444)-B-3-3-3-1-1/CL107252)DH10-B-2-B-5
37	CKH160215	(CML539/CML442)//(((KU1403 x 1368)-7-2-1-1-B-B/CML444)-B-3-3-3-1-1/CL107252)DH10-B-2-B-5
38	CKH160216	(CML545/CML548)//(((KU1403 x 1368)-7-2-1-1-B-B/CML444)-B-3-3-3-1-1/CL107252)DH10-B-2-B-5
39	CKH160217	(CZL0919/CZL0710)//(((KU1403 x 1368)-7-2-1-1-B-B/CML444)-B-3-3-3-1-1/CL107252)DH10-B-2-B-5
40	CKH160218	(CML541/CZL0718)//(((KU1403 x 1368)-7-2-1-1-B-B/CML444)-B-3-3-3-1-1/CL107252)DH10-B-2-B-5
41	CKH160219	(CML546/CZL0718)//(((KU1403 x 1368)-7-2-1-1-B-B/CML444)-B-3-3-3-1-1/CL107252)DH10-B-2-B-5
42	CKH160220	(CML540/CML539)//(((KU1403 x 1368)-7-2-1-1-B-B/CML444)-B-3-3-3-1-1/CL107252)DH10-B-2-B-8
43	CKH160221	(CML539/CML442)//(((KU1403 x 1368)-7-2-1-1-B-B/CML444)-B-3-3-3-1-1/CL107252)DH10-B-2-B-8
44	CKH160222	(CML545/CML548)//(((KU1403 x 1368)-7-2-1-1-B-B/CML444)-B-3-3-3-1-1/CL107252)DH10-B-2-B-8
45	CKH160223	(CZL0919/CZL0710)//(((KU1403 x 1368)-7-2-1-1-B-B/CML444)-B-3-3-3-1-1/CL107252)DH10-B-2-B-8
46	CKH160224	(CML541/CZL0718)//(((KU1403 x 1368)-7-2-1-1-B-B/CML444)-B-3-3-3-1-1/CL107252)DH10-B-2-B-8
47	CKH160225	(CML546/CZL0718)//(((KU1403 x 1368)-7-2-1-1-B-B/CML444)-B-3-3-3-1-1/CL107252)DH10-B-2-B-8
48	CKH160226	(CML540/CML539)//((87TZBSR-140-1-1-#/TZEESRW1-B1/EECOMP./Katumani/KATUMANI10-6-3/ECA-EE-POP1)-B-B-6/CML445)-12-1-1-6-2-1-1-2-B-B-B
49	CKH160227	(CML539/CML442)//((87TZBSR-140-1-1-#/TZEESRW1-B1/EECOMP./Katumani/KATUMANI10-6-3/ECA-EE-POP1)-B-B-6/CML445)-12-1-1-6-2-1-1-2-B-B-B

Table 2A. Names of the maize hybrids and their pedigrees

Entry	Hybrid Code	Pedigree
50	CKH160228	(CML545/CML548)//((87TZBSR-140-1-1-#/TZEESRW1-B1/EECOMP./Katumani/KATUMANI10-6-3/ECA-EE-POP1)-B-B-6/CML445)-12-1-1-6-2-1-1-2-B-B-B
51	CKH160229	(CZL0919/CZL0710)//((87TZBSR-140-1-1-#/TZEESRW1-B1/EECOMP./Katumani/KATUMANI10-6-3/ECA-EE-POP1)-B-B-6/CML445)-12-1-1-6-2-1-1-2-B-B-B
52	CKH160230	(CML541/CZL0718)//((87TZBSR-140-1-1-#/TZEESRW1-B1/EECOMP./Katumani/KATUMANI10-6-3/ECA-EE-POP1)-B-B-6/CML445)-12-1-1-6-2-1-1-2-B-B-B
53	CKH160231	(CML546/CZL0718)//((87TZBSR-140-1-1-#/TZEESRW1-B1/EECOMP./Katumani/KATUMANI10-6-3/ECA-EE-POP1)-B-B-6/CML445)-12-1-1-6-2-1-1-2-B-B-B
54	CKH160232	(CML540/CML539)//(ZEWAc1F2-80-1-1-B-1-B*4-B/CML445)-B-3-1-2-5-2-B-B
55	CKH160233	(CML539/CML442)//(ZEWAc1F2-80-1-1-B-1-B*4-B/CML445)-B-3-1-2-5-2-B-B
56	CKH160234	(CML545/CML548)//(ZEWAc1F2-80-1-1-B-1-B*4-B/CML445)-B-3-1-2-5-2-B-B
57	CKH160235	(CZL0919/CZL0710)//(ZEWAc1F2-80-1-1-B-1-B*4-B/CML445)-B-3-1-2-5-2-B-B
58	CKH160236	(CML541/CZL0718)//(ZEWAc1F2-80-1-1-B-1-B*4-B/CML445)-B-3-1-2-5-2-B-B
59	CKH160237	(CML546/CZL0718)//(ZEWAc1F2-80-1-1-B-1-B*4-B/CML445)-B-3-1-2-5-2-B-B
60	CKH160238	(CML540/CML539)//(ZEWAc1F2-80-1-1-B-1-B*4-B/CML445)-B-3-1-1-1-2-B-B
61	CKH160239	(CML539/CML442)//(ZEWAc1F2-80-1-1-B-1-B*4-B/CML445)-B-3-1-1-1-2-B-B
62	CKH160240	(CML545/CML548)//(ZEWAc1F2-80-1-1-B-1-B*4-B/CML445)-B-3-1-1-1-2-B-B
63	CKH160241	(CZL0919/CZL0710)//(ZEWAc1F2-80-1-1-B-1-B*4-B/CML445)-B-3-1-1-1-2-B-B
64	CKH160242	(CML541/CZL0718)//(ZEWAc1F2-80-1-1-B-1-B*4-B/CML445)-B-3-1-1-1-2-B-B

Table 2A. Names of the maize hybrids and their pedigrees

Entry	Hybrid Code	Pedigree
65	CKH160243	(CML546/CZL0718)//(ZEWAc1F2-80-1-1-B-1-B*4-B/CML445)-B-3-1-1-1-2-B-B
66	CKH160244	(CML540/CML539)//((87TZBSR-140-1-1-#/TZEESRW1-B1/EECOMP./Katumani/KATUMANI10-6-3/ECA-EE-POP1)-B-B-6/CML445)-12-1-1-6-2-1-1-3-B-B-B
67	CKH160245	(CML539/CML442)//((87TZBSR-140-1-1-#/TZEESRW1-B1/EECOMP./Katumani/KATUMANI10-6-3/ECA-EE-POP1)-B-B-6/CML445)-12-1-1-6-2-1-1-3-B-B-B
68	CKH160246	(CML545/CML548)//((87TZBSR-140-1-1-#/TZEESRW1-B1/EECOMP./Katumani/KATUMANI10-6-3/ECA-EE-POP1)-B-B-6/CML445)-12-1-1-6-2-1-1-3-B-B-B
69	CKH160247	(CZL0919/CZL0710)//((87TZBSR-140-1-1-#/TZEESRW1-B1/EECOMP./Katumani/KATUMANI10-6-3/ECA-EE-POP1)-B-B-6/CML445)-12-1-1-6-2-1-1-3-B-B-B
70	CKH160248	(CML541/CZL0718)//((87TZBSR-140-1-1-#/TZEESRW1-B1/EECOMP./Katumani/KATUMANI10-6-3/ECA-EE-POP1)-B-B-6/CML445)-12-1-1-6-2-1-1-3-B-B-B
71	CKH160249	(CML546/CZL0718)//((87TZBSR-140-1-1-#/TZEESRW1-B1/EECOMP./Katumani/KATUMANI10-6-3/ECA-EE-POP1)-B-B-6/CML445)-12-1-1-6-2-1-1-3-B-B-B
72	CKH160250	(CML540/CML539)//((87TZBSR-140-1-1-#/TZEESRW1-B1/EECOMP./Katumani/KATUMANI10-6-3/ECA-EE-POP1)-B-B-6/CML445)-12-1-1-6-2-1-1-4-B-B-B
73	CKH160251	(CML539/CML442)//((87TZBSR-140-1-1-#/TZEESRW1-B1/EECOMP./Katumani/KATUMANI10-6-3/ECA-EE-POP1)-B-B-6/CML445)-12-1-1-6-2-1-1-4-B-B-B
74	CKH160252	(CML545/CML548)//((87TZBSR-140-1-1-#/TZEESRW1-B1/EECOMP./Katumani/KATUMANI10-6-3/ECA-EE-POP1)-B-B-6/CML445)-12-1-1-6-2-1-1-4-B-B-B

Table 2A. Names of the maize hybrids and their pedigrees

Entry	Hybrid Code	Pedigree
75	CKH160253	(CZL0919/CZL0710)//((87TZBSR-140-1-1-#/TZEESRW1-B1/EECOMP./Katumani/KATUMANI10-6-3/ECA-EE-POP1)-B-B-6/CML445)-12-1-1-6-2-1-1-4-B-B-B
76	CKH160254	(CML541/CZL0718)//((87TZBSR-140-1-1-#/TZEESRW1-B1/EECOMP./Katumani/KATUMANI10-6-3/ECA-EE-POP1)-B-B-6/CML445)-12-1-1-6-2-1-1-4-B-B-B
77	CKH160255	(CML546/CZL0718)//((87TZBSR-140-1-1-#/TZEESRW1-B1/EECOMP./Katumani/KATUMANI10-6-3/ECA-EE-POP1)-B-B-6/CML445)-12-1-1-6-2-1-1-4-B-B-B
78	CKH160256	(CML540/CML539)//S5252Z/CML445*2-B-116-2-1-1
79	CKH160257	(CML539/CML442)//S5252Z/CML445*2-B-116-2-1-1
80	CKH160258	(CML545/CML548)//S5252Z/CML445*2-B-116-2-1-1
81	CKH160259	(CZL0919/CZL0710)//S5252Z/CML445*2-B-116-2-1-1
82	CKH160260	(CML541/CZL0718)//S5252Z/CML445*2-B-116-2-1-1
83	CKH160261	(CML546/CZL0718)//S5252Z/CML445*2-B-116-2-1-1
84	CKH160262	(CML540/CML539)//S5252Z/CML445*2-B-173-2-1-1
85	CKH160263	(CML539/CML442)//S5252Z/CML445*2-B-173-2-1-1
86	CKH160264	(CML545/CML548)//S5252Z/CML445*2-B-173-2-1-1
87	CKH160265	(CZL0919/CZL0710)//S5252Z/CML445*2-B-173-2-1-1
88	CKH160266	(CML541/CZL0718)//S5252Z/CML445*2-B-173-2-1-1
89	CKH160267	(CML546/CZL0718)//S5252Z/CML445*2-B-173-2-1-1

Table 2A. Names of the maize hybrids and their pedigrees

Entry	Hybrid Code	Pedigree
90	CKH160268	(CML540/CML539)//S6544Z/CML445*2-B-10-2-1-1
91	CKH160269	(CML539/CML442)//S6544Z/CML445*2-B-10-2-1-1
92	CKH160270	(CML545/CML548)//S6544Z/CML445*2-B-10-2-1-1
93	CKH160271	(CZL0919/CZL0710)//S6544Z/CML445*2-B-10-2-1-1
94	CKH160272	(CML541/CZL0718)//S6544Z/CML445*2-B-10-2-1-1
95	CKH160273	(CML546/CZL0718)//S6544Z/CML445*2-B-10-2-1-1
96	CKH160274	(CML540/CML539)//S6544Z/CML445*2-B-10-3-1-1
97	CKH160275	(CML539/CML442)//S6544Z/CML445*2-B-10-3-1-1
98	CKH160276	(CML545/CML548)//S6544Z/CML445*2-B-10-3-1-1
99	CKH160277	(CZL0919/CZL0710)//S6544Z/CML445*2-B-10-3-1-1
100	CKH160278	(CML541/CZL0718)//S6544Z/CML445*2-B-10-3-1-1
101	CKH160279	(CML546/CZL0718)//S6544Z/CML445*2-B-10-3-1-1
102	CKH160280	(CML540/CML539)//CML445
103	CKH160281	(CML539/CML442)//CML445
104	CKH160282	(CML545/CML548)//CML445
105	CKH160283	(CZL0919/CZL0710)//CML445
106	CKH160284	(CML541/CZL0718)//CML445

Table 2A. Names of the maize hybrids and their pedigrees

Entry	Hybrid Code	Pedigree
107	CKH160285	(CML546/CZL0718)//CML445
108	DHO4	Check
109	DK8031	Check
110	DUMA43	Check
111	P2849W	Check
112	SAWA-DSLH103	Check
113	WH507	Check
114	PAN 4M-19	Check
115	Local Check-1: 3041	Check
